# Current Perspective of Hydrogen Sulfide as a Novel Gaseous Modulator of Oxidative Stress in Glaucoma

**DOI:** 10.3390/antiox10050671

**Published:** 2021-04-26

**Authors:** Yuan Feng, Verena Prokosch, Hanhan Liu

**Affiliations:** Department of Ophthalmology, Faculty of Medicine and University Hospital of Cologne, University of Cologne, 50937 Cologne, Germany; yuanfeng.dr@gmail.com (Y.F.); verena.prokosch-willing@uk-koeln.de (V.P.)

**Keywords:** hydrogen sulfide, oxidative stress, glaucoma, neurodegeneration

## Abstract

Glaucoma is a group of diseases characterized by the progressive loss of retinal ganglion cells and their axons. Elevated intraocular pressure (IOP) is the main clinical manifestation of glaucoma. Despite being in the focus of the studies for decades, the characteristic and the exact pathology of neurodegeneration in glaucoma remains unclear. Oxidative stress is believed to be one of the main risk factors in neurodegeneration, especially its damage to the retinal ganglion cells. Hydrogen sulfide (H_2_S), the recently recognized gas signaling molecule, plays a pivotal role in the nervous system, vascular system, and immune system. It has also shown properties in regulating oxidative stress through different pathways in vivo. In this review, we summarize the distribution and the properties of H_2_S within the eye with an emphasis on its role in modulating oxidative stress in glaucoma.

## 1. Introduction

Glaucoma is a group of diseases characterized by a combination of progressive optic nerve damage and loss of visual function, with pathologically elevated intraocular pressure (IOP) being the main risk factor [1]. In clinical practice, controlling the IOP is the one and only treatment, but it usually does not halt the progression of glaucoma and the damage to the optic nerve [2]. This situation also reflects the fact that the pathogenesis of glaucoma is multifactorial and extremely complex. It remains to be thoroughly studied, however, certain pathological processes are identified to play a role in the progress of glaucoma, such as excessive oxidative stress, inflammatory reactions, and accumulation of protein mutations [3,4,5,6]. Aging is a well-known risk factor for neurodegenerative diseases as well as for glaucoma. The incidence of glaucoma worldwide increases three-fold within ageing population every decade [7] and the number of glaucoma patients is expected to be 112 million by 2040 [8]. Oxidative stress is proposed as a key modulator in ageing and in neurodegenerative diseases [9]. There is hypothesis that age-related neuronal functional losses are due to the accumulation of oxidative stress [10]. An overall increase of oxidative stress markers is also detected in glaucoma patients [11].

The reactive oxygen species (ROS) is essential for normal cellular function. When the production of ROS exceeds the antioxidant capacity of the cells, they pose a constant threat to the cell, a process known as oxidative stress [6]. The higher concentration and long-time exposure of oxidative stress cause damage to cellular macromolecules such as DNA, lipids, and proteins, ultimately resulting in necrosis and apoptotic cell death [9]. The characters of neurodegenerative diseases are apoptosis/necrosis and dysfunction of neuronal cells, resulting in neurodegenerative changes due to nerve cell dysfunction [9,12,13,14]. In glaucoma, oxidative stress harms multiple ocular tissues via different mechanisms, which comprising stimulating apoptotic and inflammatory pathways at the trabecular meshwork level and promoting retinal ganglion cell apoptosis and glial dysfunction [15,16,17,18]. These studies contributed to our understanding of oxidative stress in glaucoma. However, the cellular mechanisms of oxidative damage in the eye and the retinal ganglion cells (RGCs), as well as the possible regulatory mechanisms in response to oxidative stress remain to be further elucidated.

H_2_S has been recently recognized to be a third gas signaling molecular of comparable importance to nitric oxide (NO) and carbon monoxide (CO) [19,20]. H_2_S plays a significant role in the human nervous system, vascular system, and immune system at a certain range of concentration [21,22,23]. The concentration of H_2_S in the peripheral blood is generally 30–300 Mm [24], while the physiological concentration of H_2_S in the brain is up to three times that in serum [25,26].

In the central nervous system (CNS), H_2_S is present in high concentrations and has shown protective effects on neurons by modulating oxidative stress and inflammatory responses, anti-apoptosis as well as acting as a vasculoprotective factor [27,28,29,30]. In cultured cells, H_2_S protects primary cortical neurons from oxidative stress induced by glutamate by increasing the production of antioxidant glutathione [31]. In the peripheral nervous system, H_2_S has been proven to protect RGCs, the neuron that is selected to die in glaucoma, in different pathological conditions, such as diabetic retinopathy, *N*-methyl-d-aspartic acid (NMDA)-induced neurotoxicity, and ischemic condition [16,19,20]. There has been an increasing interest in studying the role of H_2_S in neurodegeneration in glaucoma. We documented in our previous study that the administration of exogenous H_2_S protects RGC against different glaucomatous injuries in vitro and in vivo [21]. The underlying mechanism was partly attributed to its capability of vasorelaxation, anti-oxidative stress, neuroendocrine regulation, and inflammation suppression [22,23,24]. Although it is yet inconclusive as to through which mechanism exerts H_2_S its protective effects in glaucoma. By all counts, H_2_S plays a pivotal role in protecting RGCs and their axons against neurodegeneration.

This review aims to firstly summarize the generation and distribution of H_2_S in the eye, and secondly to further explore the interaction of H_2_S and oxidative stress in neurodegeneration in glaucoma, with a focus on the diverse underlying pathways.

## 2. Generation and Distribution of H_2_S in Ocular Tissues

There are two main production pathways of H_2_S in mammalian cells, enzymatic and non-enzymatic pathways [32]. Enzymatic pathways account for the central part. The enzymes known to participate in the enzymatic pathway of production of H_2_S are mainly cystathionine-γ-synthase (CSE), cystathionine-β-lyase (CBS), 3-mercapto-methylthio pyruvate aminotransferase (3MST), and cysteine aminotransferase (CAT) [32,33]. Generally, endogenous H_2_S is derived from the desulfurization of cysteine or homocysteine by the enzymes CSE and CBS. Different enzymes are expressed in different systems. CBS expression is significant in the brain as the primary physiological source of H_2_S in the central nervous system [34]. Robert et al. documented that CBS proteins are widely present in the adult rat brain and are most strongly expressed in the Purkinje cell layer and hippocampus [34]. CSE is abundantly expressed in the respiratory system and cardiovascular system [20]. Both CBS and CSE are only localized in the cytoplasm, while 3MST and CAT exist in both mitochondria and cytoplasm. Recently, it has also been demonstrated that H_2_S can also be produced from d-cysteine via d-amino acid oxidase (DAO) along with 3MST [35]. However, production through this pathway is limited as DAO exists exclusively in the brain and kidneys [35]. On the other hand, the non-enzymatic pathways of endogenous H_2_S are produced in erythrocytes through the glucose oxidation pathway [19]. Moreover, this pathway can be stimulated by increased oxidative stress and hyperglycemia to produce more H_2_S [32].

**Figure 1:**
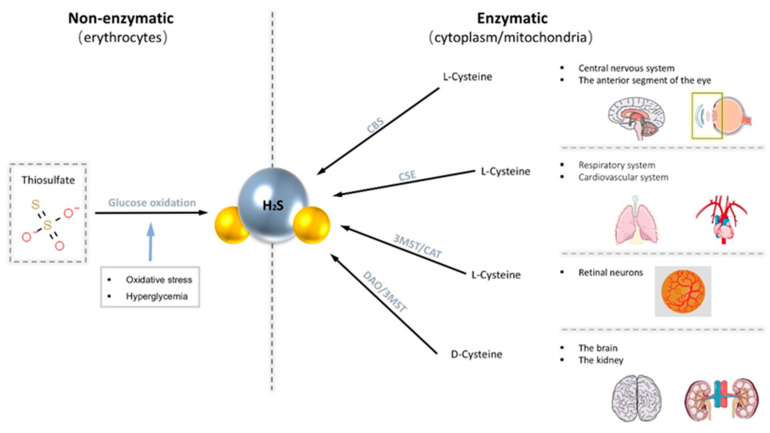
Endogenous H_2_S is produced through two main pathways, the non-enzymatic pathway and the enzymatic pathway. The non-enzymatic pathway is produced in erythrocytes via the glucose oxidation pathway. The enzymatic pathway promotes the production of H_2_S from cysteine with four common enzymes (cystathionine-β-lyase (CBS), cystathionine-γ-synthase (CSE), 3-mercapto-methylthio pyruvate aminotransferase (3MST), and cysteine aminotransferase (CAT)). These enzymes are distributed in various tissues of the body.

These H_2_S-productive enzymes are widely distributed in specific tissues in the eye, predominately in the cornea and retina [33,36,37,38,39]. CBS is present in various ocular tissues, including conjunctiva, cornea, iris, lens, retina, and optic nerve, but not in vitreous humor [36,40]. CBS is abundant in anterior segments throughout the lifespan, and its abundance within the retina increases with age [33,36]. The presence of CBS and CSE has also been traced in all three layers of canine, non-human primate, and human retina: photoreceptors, outer plexiform layer (OPL), and notably in the ganglion cells layer/nerve fiber layer (GCL/NFL) [41]. 3MST/CAT pathway is the primary way to produce H_2_S in the mammalian retina as both 3MST and CAT are located in the retinal neurons [36]. Our previous study has shown that H_2_S produced by the 3-MST pathway increases after seven weeks of IOP elevation in a glaucoma animal model [42] (Figure 1).

## 3. The Anti-Oxidative Properties of H_2_S in Glaucoma

### 3.1. Reducing Intraocular Pressure (IOP)

Elevated intraocular pressure is the hallmark of the development of glaucoma, and it is also considered one of the main causes of damage to retinal neurons and optic nerves.

The production of aqueous humor (AH) in the ciliary body and the unobstructed flow of various AH outflow pathways are important factors in determining stable IOP. 

The autonomic regulation of the blood vessels of the ciliary body and the ciliary epithelium is crucial in the production of AH, while episcleral blood vessels are in the outflow of AH [43]. In a study on isolated superfused bovine iris-ciliary bodies, all three H_2_S producing substances (ACS67, a hybrid of latanoprost and an H_2_S-donating moiety, l-cysteine, a substrate for endogenous production of H_2_S, and GYY4137, Morpho-lin-4ium-methoxyphenyl-morpholino-phosphinodithioate, a slow-releasing H_2_S donor) inhibited sympathetic neurotransmission in the iris-ciliary bodies through the mediation of K_ATP_ channels [44]. Notably, the reduction of sympathetic neurotransmission in the anterior uvea relaxes the iris ciliary muscle and diastolic ocular vascular smooth muscle, indirectly acting to lower IOP [40,45]. In the isolated porcine iris ciliary body, both H_2_S inhibited norepinephrine release from sympathetic nerve terminals in the eye which promotes the atrial outflow [40,46]. Its ability in modulating autonomic nerves indicates that H_2_S has a critical influence on the anterior uveal tissue as well as the production and the outflow of AH.

Other than the episcleral blood vessels, the regulatory processes of the trabecular meshwork are also pivotal in regulating conventional aqueous humor outflow from the anterior chamber [43]. The trabecular meshwork (TM) is a key region for the initiation of glaucoma. Excessive ROS alters both TM motility and cytoarchitecture [18,47,48]; apoptotic TM cells and affected TM epithelial cells impair aqueous outflow [49,50,51], which leads to a failure to control IOP, a hallmark in the progress of glaucoma [52]. H_2_S is shown to facilitate the dynamic equilibrium of AH and stabilize IOP by increasing autonomic regulation in ocular anterior segments, reduce the cell volume of trabecular meshwork and relax the iris smooth muscle [33,45,53,54].

Elevated IOP leads to increased oxidative stress in the eye. Documented by Gericke et al., elevated levels of ROS were detected in the RGC layer and retinal vasculature in a mouse model with elevated IOP [55].

By regulating AH dynamic, H_2_S and its donors can effectively modulate the oxidative stress caused by elevated IOP.

### 3.2. Ocular Hemodynamic Changes

The retina, as an extension of the CNS, has an equally high demand for oxygen and other metabolites as the brain [56]. Lack of oxygen can lead to eye diseases such as diabetic retinopathy and glaucoma [57,58]. However, the vascular density in the inner retina is limited due to the optical function of the eye, which leads to an unstable vascular oxygen supply [59]. This feature renders the inner retina, especially the ganglion cell layer and the nerve fiber layer, extremely vulnerable to changes in ocular hemodynamic. It has been discussed over a decade, that hemodynamic change plays a role in glaucomatous neuropathy [60,61]. Studies have provided evidence that compared with healthy individuals, the blood flow in the eyes of glaucoma patients is significantly reduced, furthermore, the blood flow in the optic papilla region is reduced more obviously [62,63,64]. Furthermore, this was also demonstrated in our previous study in an in vivo glaucoma animal model, elevated IOP leads to shrinking of retinal vascular caliber [65], which limits the blood flow and oxygen supply in the retina and consequently promotes the production and accumulation of ROS [66]. Morphological and functional changes of the vascular play a central role in hemodynamic changes.

Like NO, H_2_S has similar effects on blood vessels. A low concentration of H_2_S relaxes the vascular smooth muscle [32]. However, it is demonstrated that NO mainly acts on large blood vessels, while H_2_S has a more significant effect on small blood vessels [32]. This difference may be related to H_2_S and blood oxygen concentration. According to several reports, H_2_S can exert a dilation effect on blood vessels at higher than physiological oxygen levels but lead to the opposite effect at lower than physiological oxygen levels [67]. The human eye is surrounded by small blood vessels and the partial pressure of oxygen in small blood vessels is low, therefore H_2_S may have a more substantial role in vasodilation in the ocular vasculature.

Slow releasing H_2_S donors, 4-methoxyphenyl)pyrrolidin-1-ylphosphinodithioc acid (AP67) and 4-methoxyphenyl)piperidin-1-ylphosphinodithioc acid (AP72) have been demonstrated to have vasodilatory effects on isolated bovine posterior ciliary arteries induced by adrenergic receptor agonists [68]. Moreover, this process may be dependent on the biosynthesis of endogenous NO and the action of K_ATP_ channels [68]. In our previous study, we also observed that intravitreal injection of GYY4137 into mice increased the caliber of retinal vessels and significantly improved blood flow, which is beneficial in improving retinal perfusion and subsequently promotes RGC survival over the long term [42]. By expanding blood vessels, H_2_S improves blood flow and oxygen and metabolite to the retina as well as stabilizes retina hemodynamics, therefore modulates the oxidative stress in the retina in glaucoma.

### 3.3. Inhibition of Neurodegeneration in Glaucoma

The neurodegenerative changes in glaucoma are mainly manifested by progressive retinal ganglion cell death. The changes can be contributed to the absence of certain neurotrophic factors, intracellular and extracellular toxicity of glutamate, and stimulation of external factors [6,31]. Such neurodegenerative changes become more pronounced with age, accumulation of ROS, and reduction of antioxidant substances. In a mouse model of Alzheimer’s disease (AD), H_2_S was found to interfere with the production of amyloid β-protein (Aβ), which affects the development of AD, and inhibits Aβ-induced neuronal apoptosis [69,70]. Similarly, in an animal model of Parkinson’s disease (PD), H_2_S was found to act as an antioxidant to counteract the neurotoxic effects induced by 6-hydroxydopamine and to have a neuroprotective effect [71]. At the same time, in a clinical study that the H_2_S was significantly lower in the plasma of AD patients than in healthy controls [69,72,73]. These studies may infer that H_2_S plays a role in neurodegeneration in AD and protects neurological function and prevents neurodegeneration in animal models of PD and AD through anti-apoptotic, anti-inflammatory, and antioxidant pathways. However, the exact pharmacology of H_2_S in the clinical treatment of neurodegenerative diseases requires further research. In the eye, H_2_S has also been found to protect RGCs against glaucomatous damages caused by elevated IOP and oxidative stress both in vivo and in vitro in animal models [42].

**Figure 2:**
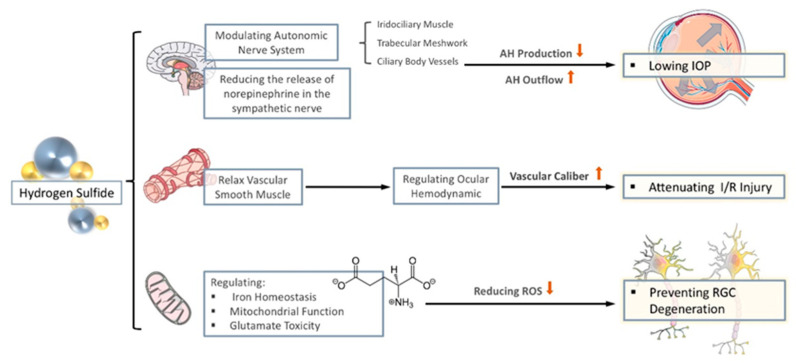
The anti-oxidative properties of H_2_S in glaucoma. The effect of H_2_S on glaucoma is mainly reflected in three aspects. (1) By regulating the autonomic nerves and reducing the release of sympathetic nerves, it reduces the production of aqueous humor (AH) and promotes the flow of AH to lowering intraocular pressure (IOP). (2) Through the relaxation of vascular smooth muscles, stabilize intraocular perfusion and reduce ischemia-reperfusion injury. (3) By regulating iron homeostasis, regulating mitochondrial function, and reducing the toxicity of glutamate, it reduces the generation of ROS to prevent nerve degenerative changes.

Although the mechanism of H_2_S in protecting against glaucomatous optic nerve damage is to be thoroughly investigated, there are traces to follow, that H_2_S may regulate oxidative stress and protect against neurodegenerative changes in glaucoma by regulating iron homeostasis, changing mitochondrial dysfunction, and attenuate glutamate neurotoxicity. In the following chapters, we aim to summarize the established mechanistic link between H_2_S and oxidative stress in neurodegeneration from recently published studies (see Figure 2).

#### 3.3.1. Regulation of Iron Homeostasis

Iron homeostasis plays a pivotal role in oxidative damage. Excess iron promotes the generation of damaging hydroxyl groups from oxidation reaction products, thus exacerbating oxidative stress [74,75]. Current studies have found that iron metabolism is critical for neurotransmitter production and myelin synthesis [76]. In a number of studies on neurodegenerative diseases, it has been found that increased levels of iron in the brains of animals and human produce significant cognitive impairment [77,78,79,80]. Furthermore, MRI scans have shown abnormal aggregation of iron in the hippocampus in the brains of post-mortem AD patients and AD mouse models [81,82]. It has also been shown in patients with PD, total iron concentrations in the substantia nigra are increased [71]. These evidences suggest that altered iron levels in the brain are associated with neuronal dysfunction and the pathology of neurodegenerative disease in CNS in both animal and human. Increased iron level has also been documented in not only various glaucoma models but also in AH of glaucoma patients [83,84]. As research continues to progress, alteration in iron homeostasis has been also connected to intracellular glutamate production and secretion, glutathione (GSH) synthesis, and hypoxia-inducible factor-1 (HIF-1) activity, yet all of these pathways are implicated in the pathogenesis of glaucoma [85]. These results indicate that iron homeostasis may also play a role in neurodegenerative changes in glaucoma.

The majority of iron in cells is located in the protoporphyrin ring of heme. The massive release of heme can lead to severe oxidative stress and promote apoptosis [74]. Maintaining heme release can effectively maintain iron homeostasis and control redox reactions. Under both hyperoxia conditions and ischemia-reperfusion injury, H_2_S has been shown to modulate the heme release, by interacting with heme oxygenase and biliverdin reductase A, respectively, while increasing the capacity of antioxidant production [76,86,87].

Another regulating iron homeostasis, H_2_S may also play a crucial role in the iron-sulfide (Fe-S) cluster. Fe-S cluster is essential for retinal physiology and pathology [88,89]. Fe-S cluster is required for many essential processes in the cell, including catalysis, iron regulation, DNA repair, ribosome biogenesis, and tRNA modifications [90,91]. Fe-S clusters are also involved in many other vital processes in mitochondria. They not only donate electrons to the respiratory chain, but also constitute the respiratory protein complexes I, II, and III [92]. Defects in Fe-S cluster synthesis in mitochondria are generally not considered to be re-sponsible for all neurodegenerative diseases, but such defects can affect the normal function of the central nervous system, for instance, in X-linked sideroblastic anemia and mitochondrial encephalopathy, Fe-S cluster synthesis is believed to play a role in their pathogenesis [92,93,94]. Since both components (iron and sulfur) are toxic, Fe-S cluster assembly must therefore be both efficient and tightly regulated [95]. Glutaredoxin-3 (Glrx3) is an important factor in the assembly of Fe-S clusters [96]. H_2_S is shown to regulate intracellular Glrx3 levels, thereby maintaining iron homeostasis and regulating the redox state in cells [76].

#### 3.3.2. Changes in Mitochondrial Function

Mitochondria are the most important energy-producing organelles in human cells. It produces energy through a variety of ways, which coordinate with each other to maintain the best energy state in the cell [97]. When mitochondria generate energy, they also generate many ROS, including hydrogen peroxide (H_2_O_2_), superoxide (O_2_^•−^), and hydroxyl ion (^•^OH) [98]. Mitochondrial dysfunction leads not only to reduced ATP production but also to excessive ROS production, impaired calcium buffering, mitochondrial metal allostasis, and activation of mitochondria-dependent apoptosis [99]. In recent years, mitochondrial disorders have been considered to be an important factor in the development of age-related neurodegenerative diseases such as AD, PD, amyotrophic lateral sclerosis (ALS), and Freidriech ataxia (FRDA) [100]. This is mainly associated with DNA defects in the mitochondria, abnormal mitochondrial enzyme activity, and abnormal expression of mitochondrial genes. These abnormalities in mitochondrial function leading to the development of neurodegenerative diseases have been studied in many animal studies and in post-mortem autopsies of patients [101,102,103]. Worth noting, is that not all neuronal cells are susceptible to mitochondrial dysfunction. In Leber’s hereditary optic neuropathy, a classical mitochondrial disease, only RGCs are selected to die while other neuronal population remains unaffected. Retinal ganglion cells have a high energy requirement, which can be reflected by the high cytochrome c oxidase activity [104], they are highly susceptible to mitochondrial dysfunction. Therefore, mitochondrial dysfunction is likely to play a role in glaucoma [97,98,99].

The intracellular production of ATP relies heavily on the high efficiency of the mitochondrial electron transport chain (ETC). However, the nature of its electron exchange leads to its susceptibility to side effects, such as with molecular oxygen, which reduces the production of ATP [105,106]. H_2_S is a “double-edged sword” in the mitochondrial energy production process and has different effects on the ETC at different physiological concentrations. At low concentrations, H_2_S is mainly a substrate for ETC, providing electrons to ETC at the ubiquinone level [107,108]. It stimulates oxidative phosphorylation and promotes the production of ATP. However, at higher concentrations, the inhibitory effect of H_2_S on cyclooxygenase (COX)is predominated, thus reducing ATP production and decreasing ROS generation [107,108]. Furthermore, H_2_S also modulates the production of ATP under different physio pathological conditions, thereby reducing ROS production by ETC and protecting cells from oxidative stress. H_2_S reduces ATP production by up-regulating coupling protein (UCP)-2 and down-regulating protein expression of COX I and II subunits [109,110].

In research of altered protein expression in the retina in a mouse model of acute IOP H_2_S was found to limit ROS production by inhibiting mitochondrial oxygen consumption and leading to increased intracellular oxygen tension, possibly through upregulating HIF1-α levels and decreasing mitochondrial oxidative phosphorylation levels [76]. This research also found that H_2_S promotes the production of ketone bodies, which can replace glucose as an energy source and enhance retinal resistance to oxidative stress, thereby reducing neuronal damage [76].

In addition, mutations in mitochondrial DNA (mtDNA) also lead to alterations in ETC [99]. Since mtDNA lacks protective proteins against oxygen radicals, it is susceptible to ROS and leads to mutations [111,112]. However, AP39, an H_2_S donor, is proven to prevent the destruction of mtDNA by glucose oxidase and plays an antioxidant and cytoprotective role [113]. It has also been shown that H_2_S maintains the transcriptional process of mtDNA by controlling the mitochondrial transcription factor A (TFAM) [114].

#### 3.3.3. Effect on Glutathione Production Pathway

Glutathione is a tripeptide composed of glutamic acid, cysteine, and glycine. It is also one of the most common antioxidants in the human body. The sulfhydryl group on cysteine is the active group of glutathione, which is readily combined with free radicals induced through oxidative stress and has anti-neurotoxic effects, thus protecting neurons from oxidative damage [115,116,117]. It is shown that knockout of the gene for an enzyme affecting glutathione synthesis produced spontaneous apoptosis of RGC and damage to the optic nerve in mice without elevated IOP [118]. This demonstrates that glutathione has a pivotal role in the mechanism of RGC loss independent elevated IOP.

Glutathione synthesis is strongly influenced by extracellular glutamate. Cystine is the main source of cysteine (required for intracellular glutathione synthesis) and since glutamate and cystine share the same amino acid transport protein, high concentrations of extracellular glutamate compete with cystine to prevent intracellular glutathione synthesis [31,119,120]. However, H_2_S effectively prevents this competition, thus restoring normal cystine transport and promoting intracellular glutathione synthesis [31]

Moreover, γ-glutamylcysteine synthase (γ-GCS) and γ-glutamylcysteine (γ-GC) are key enzymes in the synthesis of glutathione, and their activities directly affect the intracellular concentration of glutathione. Kimura et al. documented a two-fold increase in γ-GC levels in isolated rat neuronal cells after culturing with additional H_2_S donors for 2 h. Whereas in the control group, intracellular γ-GC showed a decreasing trend over time [31]. Promoting the intracellular levels of enzymes involved in glutathione synthesis might be part of the mechanism of how H_2_S increases intracellular glutathione.

**Figure 3:**
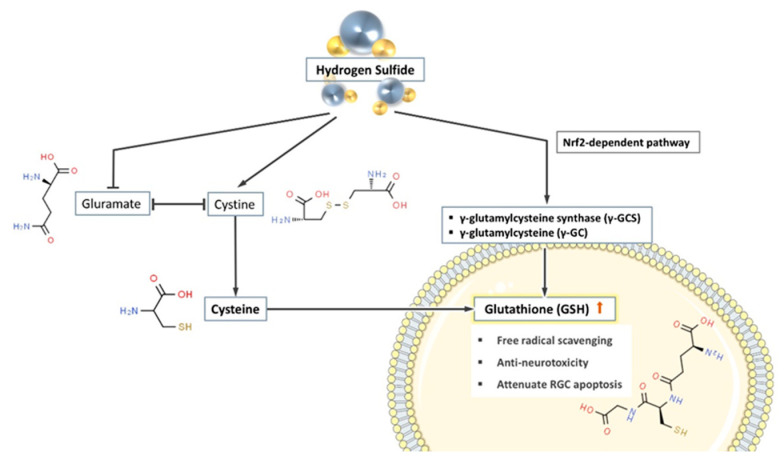
H_2_S promotes the synthesis of glutathione in cells through three pathways. (1) H_2_S suppresses the extracellular competition between glutamate and cystine, therefore promotes cystine (the substrate of glutathione (GSH) synthesis) entering the cells. (2) H_2_S promotes the production of a variety of glutathione synthase and increases the concentration of GSH in the cell. (3) H_2_S indirectly promotes the synthesis of GSH through the Nrf2-dependent pathway.

At the same time, the gene expression of γ-GCS and GSH synthase can also be regulated by the nuclear factor erythroid 2–related factor 2 (Nrf2) [121]. It is reported that H_2_S donor promotes the synthesis of GSH through an Nrf2-dependent pathway [122]. In summary, H_2_S and its donors directly or indirectly promote the synthesis of glutathione in cells, thus achieving the effect of preventing neurodegenerative changes (see Figure 3).

## 4. Conclusions

H_2_S is undoubtedly an attractive candidate of potential treatment in glaucoma. It is shown in glaucoma animal models both in vitro and in vivo, H_2_S reduces intraocular oxidative stress and prevents neurodegeneration. However, the key roadblock to translational research in field of glaucoma is insufficient understanding of its pathophysiology. Therefore, there is a lack of good models. Using different glaucoma models goes some way to reflect therapeutic potential of H_2_S in glaucoma. It has to be kept in mind that the results obtained from animal studies may not be fully translatable in human patients. The pharmacology of H_2_S in glaucoma remains to be further investigated.

## Data Availability

Data is contained within the article.

## References

[1] Casson R.J., Chidlow G., Wood J.P.M., Crowston J.G., Goldberg I. (2012). Definition of glaucoma: Clinical and experimental concepts. Clin. Exp. Ophthalmol..

[2] Chang E.E., Goldberg J.L. (2012). Glaucoma 2.0: Neuroprotection, Neuroregeneration, Neuroenhancement. Ophthalmology.

[3] Huang S., Huang P., Liu X., Lin Z., Wang J., Xu S., Guo L., Leung C.K.-S., Zhong Y. (2017). Relevant variations and neuroprotecive effect of hydrogen sulfide in a rat glaucoma model. Neuroscience.

[4] Križaj D., Ryskamp D.A., Tian N., Tezel G., Mitchell C.H., Slepak V.Z., Shestopalov V.I. (2014). From Mechanosensitivity to Inflammatory Responses: New Players in the Pathology of Glaucoma. Curr. Eye Res..

[5] Biermann J., Lagreze W.A., Schallner N., Schwer C.I., Goebel U. (2011). Inhalative preconditioning with hydrogen sulfide attenuated apoptosis after retinal ischemia/reperfusion injury. Mol. Vis..

[6] Tezel G. (2006). Oxidative stress in glaucomatous neurodegeneration: Mechanisms and consequences. Prog. Retin. Eye Res..

[7] Mukesh B.N., McCarty C.A., Rait J.L., Taylor H.R. (2002). Five-year incidence of open-angle glaucoma. Ophthalmology.

[8] Quigley H.A. (2006). The number of people with glaucoma worldwide in 2010 and 2020. Br. J. Ophthalmol..

[9] Singh A., Kukreti R., Saso L., Kukreti S. (2019). Oxidative Stress: A Key Modulator in Neurodegenerative Diseases. Molecules.

[10] Castelli V., Benedetti E., Antonosante A., Catanesi M., Pitari G., Ippoliti R., Cimini A., d’Angelo M. (2019). Neuronal Cells Rearrangement During Aging and Neurodegenerative Disease: Metabolism, Oxidative Stress and Organelles Dynamic. Front. Mol. Neurosci..

[11] D’Azy C.B., Pereira B., Chiambaretta F., Dutheil F. (2016). Oxidative and Anti-Oxidative Stress Markers in Chronic Glaucoma: A Systematic Review and Meta-Analysis. PLoS ONE.

[12] Alam Z.I., Jenner A., Daniel S.E., Lees A.J., Cairns N., Marsden C.D., Jenner P., Halliwell B. (2002). Oxidative DNA Damage in the Parkinsonian Brain: An Apparent Selective Increase in 8-Hydroxyguanine Levels in Substantia Nigra. J. Neurochem..

[13] Halliwell B., Gutteridge J.M.C. (1984). Oxygen toxicity, oxygen radicals, transition metals and disease. Biochem. J..

[14] Mariani E., Polidori M., Cherubini A., Mecocci P. (2005). Oxidative stress in brain aging, neurodegenerative and vascular diseases: An overview. J. Chromatogr. B.

[15] Huang S., Huang P., Lin Z., Liu X., Xu X., Guo L., Shen X., Li C., Zhong Y. (2018). Hydrogen sulfide supplement attenuates the apoptosis of retinal ganglion cells in experimental glaucoma. Exp. Eye Res..

[16] Nita M., Grzybowski A. (2016). The Role of the Reactive Oxygen Species and Oxidative Stress in the Pathomechanism of the Age-Related Ocular Diseases and Other Pathologies of the Anterior and Posterior Eye Segments in Adults. Oxidative Med. Cell. Longev..

[17] Kruk J., Kubasik-Kladna K., Aboul-Enein H.Y. (2015). The Role Oxidative Stress in the Pathogenesis of Eye Diseases: Current Status and a Dual Role of Physical Activity. Mini Rev. Med. Chem..

[18] Saccà S., Izzotti A. (2008). Oxidative stress and glaucoma: Injury in the anterior segment of the eye. Prog. Brain Res..

[19] Wang R. (2002). Two’s company, three’s a crowd: Can H_2_S be the third endogenous gaseous transmitter?. FASEB J..

[20] Zhao W., Zhang J., Lu Y., Wang R. (2001). The vasorelaxant effect of H_2_S as a novel endogenous gaseous KATP channel opener. EMBO J..

[21] Gadalla M.M., Snyder S.H. (2010). Hydrogen sulfide as a gasotransmitter. J. Neurochem..

[22] Wu L., Wang R. (2005). Carbon Monoxide: Endogenous Production, Physiological Functions, and Pharmacological Applications. Pharmacol. Rev..

[23] Snyder S.H., Jaffrey S.R., Zakhary R. (1998). Nitric oxide and carbon monoxide: Parallel roles as neural messengers. Brain Res. Rev..

[24] Olson K.R. (2009). Is hydrogen sulfide a circulating “gasotransmitter” in vertebrate blood?. Biochim. Biophys. Acta Bioenerg..

[25] Hogg P.J. (2009). Contribution of allosteric disulfide bonds to regulation of hemostasis. J. Thromb. Haemost..

[26] Zhong G.Z., Chen F.R., Cheng Y.Q., Tang C.S., Du J.B. (2003). The role of hydrogen sulfide generation in the pathogenesis of hypertension in rats induced by inhibition of nitric oxide synthase. J. Hypertens..

[27] Gao R., Chen G., Zhang J.-Y., Ding Y.-P., Wang Z., Kong Y. (2017). Hydrogen sulfide therapy in brain diseases: From bench to bedside. Med. Gas Res..

[28] Streeter E.Y., Badoer E., Woodman O.L., Hart J.L. (2013). Effect of type 1 diabetes on the production and vasoactivity of hydrogen sulfide in rat middle cerebral arteries. Physiol. Rep..

[29] Luo Y., Liu X., Zheng Q., Wan X., Ouyang S., Yin Y., Sui X., Liu J., Yang X. (2012). Hydrogen sulfide prevents hypoxia-induced apoptosis via inhibition of an H_2_O_2_-activated calcium signaling pathway in mouse hippocampal neurons. Biochem. Biophys. Res. Commun..

[30] Li L., Bhatia M., Moore P.K. (2006). Hydrogen sulphide—A novel mediator of inflammation?. Curr. Opin. Pharmacol..

[31] Kimura Y., Kimura H. (2004). Hydrogen sulfide protects neurons from oxidative stress. FASEB J..

[32] Wang R. (2012). Physiological Implications of Hydrogen Sulfide: A Whiff Exploration That Blossomed. Physiol. Rev..

[33] Han Y., Shang Q., Yao J., Ji Y. (2019). Hydrogen sulfide: A gaseous signaling molecule modulates tissue homeostasis: Implications in ophthalmic diseases. Cell Death Dis..

[34] Robert K., Vialard F., Thiery E., Toyama K., Sinet P.-M., Janel N., London J. (2003). Expression of the Cystathionine β Synthase (CBS) Gene During Mouse Development and Immunolocalization in Adult Brain. J. Histochem. Cytochem..

[35] Kimura H. (2015). Signaling Molecules: Hydrogen Sulfide and Polysulfide. Antioxid. Redox Signal..

[36] Persa C., Osmotherly K., Chen K.C.-W., Moon S., Lou M.F. (2006). The distribution of cystathionine β-synthase (CBS) in the eye: Implication of the presence of a trans-sulfuration pathway for oxidative stress defense. Exp. Eye Res..

[37] Pong W.W., Stouracova R., Frank N., Kraus J.P., Eldred W.D. (2007). Comparative localization of cystathionine β-synthase and cystathionine γ-lyase in retina: Differences between amphibians and mammals. J. Comp. Neurol..

[38] Mikami Y., Shibuya N., Kimura Y., Nagahara N., Yamada M., Kimura H. (2011). Hydrogen Sulfide Protects the Retina from Light-induced Degeneration by the Modulation of Ca2+ Influx. J. Biol. Chem..

[39] Kulkarni M., Njie-Mbye Y.F., Okpobiri I., Zhao M., Opere C.A., Ohia S.E. (2011). Endogenous Production of Hydrogen Sulfide in Isolated Bovine Eye. Neurochem. Res..

[40] Ohia S.E., Robinson J., Mitchell L., Ngele K.K., Heruye S., Opere C.A., Njie-Mbye Y.F. (2018). Regulation of Aqueous Humor Dynamics by Hydrogen Sulfide: Potential Role in Glaucoma Pharmacotherapy. J. Ocul. Pharmacol. Ther..

[41] Badiei A., Sudharsan R., Santana E., Dunaief J.L., Aguirre G.D. (2019). Comparative localization of cystathionine beta synthases and cystathionine gamma lyase in canine, non-human primate and human retina. Exp. Eye Res..

[42] Liu H., Anders F., Thanos S., Mann C., Liu A., Grus F.H., Pfeiffer N., Prokosch-Willing V. (2017). Hydrogen Sulfide Protects Retinal Ganglion Cells Against Glaucomatous Injury In Vitro and In Vivo. Investig. Opthalmol. Vis. Sci..

[43] McDougal D.H., Gamlin P.D. (2014). Autonomic Control of the Eye. Compr. Physiol..

[44] Salvi A., Bankhele P., Jamil J.M., Kulkarni-Chitnis M., Njie-Mbye Y.F., Ohia S.E., Opere C.A. (2016). Pharmacological Actions of Hydrogen Sulfide Donors on Sympathetic Neurotransmission in the Bovine Anterior Uvea, In Vitro. Neurochem. Res..

[45] Robinson J., Okoro E., Ezuedu C., Bush L., Opere C.A., Ohia S.E., Njie-Mbye Y.F. (2017). Effects of Hydrogen Sulfide-Releasing Compounds on Aqueous Humor Outflow Facility in Porcine Ocular Anterior Segments, Ex Vivo. J. Ocul. Pharmacol. Ther..

[46] Kulkarni K.H., Monjok E.M., Zeyssig R., Kouamou G., Bongmba O.N., Opere C.A., Njie Y.F., Ohia S.E. (2009). Effect of Hydrogen Sulfide on Sympathetic Neurotransmission and Catecholamine Levels in Isolated Porcine Iris-Ciliary Body. Neurochem. Res..

[47] He Y., Ge J., Tombran-Tink J. (2008). Mitochondrial Defects and Dysfunction in Calcium Regulation in Glaucomatous Trabecular Meshwork Cells. Investig. Opthalmol. Vis. Sci..

[48] Tanwar M., Dada T., Sihota R., Dada R. (2010). Mitochondrial DNA analysis in primary congenital glaucoma. Mol. Vis..

[49] Saccà S.C., Izzotti A. (2014). Focus on molecular events in the anterior chamber leading to glaucoma. Cell. Mol. Life Sci..

[50] Alvarado J., Murphy C., Juster R. (1984). Trabecular Meshwork Cellularity in Primary Open-angle Glaucoma and Nonglaucomatous Normals. Ophthalmology.

[51] Pulliero A., Seydel A., Camoirano A., Saccà S.C., Sandri M., Izzotti A. (2014). Oxidative Damage and Autophagy in the Human Trabecular Meshwork as Related with Ageing. PLoS ONE.

[52] Wiederholt M., Thieme H., Stumpff F. (2000). The regulation of trabecular meshwork and ciliary muscle contractility. Prog. Retin. Eye Res..

[53] Monjok E.M., Kulkarni K.H., Kouamou G., McKoy M., Opere C.A., Bongmba O.N., Njie Y.F., Ohia S.E. (2008). Inhibitory action of hydrogen sulfide on muscarinic receptor-induced contraction of isolated porcine irides. Exp. Eye Res..

[54] Bucolo C., Drago F. (2011). Carbon monoxide and the eye: Implications for glaucoma therapy. Pharmacol. Ther..

[55] Gericke A., Mann C., Zadeh J.K., Musayeva A., Wolff I., Wang M., Pfeiffer N., Daiber A., Li H., Xia N. (2019). Elevated Intraocular Pressure Causes Abnormal Reactivity of Mouse Retinal Arterioles. Oxidative Med. Cell. Longev..

[56] Werkmeister R.M., Schmidl D., Aschinger G., Doblhoff-Dier V., Palkovits S., Wirth M., Garhöfer G., Linsenmeier R.A., Leitgeb R.A., Schmetterer L. (2015). Retinal oxygen extraction in humans. Sci. Rep..

[57] Wangsa-Wirawan N.D. (2003). Retinal Oxygen. Arch. Ophthalmol..

[58] Cringle S.J., Yu D.-Y. (2009). Oxygen supply and consumption in the retina: Implications for studies of retinopathy of prematurity. Doc. Ophthalmol..

[59] Joyal J.-S., Gantner M.L., Smith L.E. (2018). Retinal energy demands control vascular supply of the retina in development and disease: The role of neuronal lipid and glucose metabolism. Prog. Retin. Eye Res..

[60] Fechtner R.D., Weinreb R.N. (1994). Mechanisms of optic nerve damage in primary open angle glaucoma. Surv. Ophthalmol..

[61] Yan D.B., Coloma F.M., Metheetrairut A., Trope G.E., Heathcote J.G., Ethier C.R. (1994). Deformation of the lamina cribrosa by elevated intraocular pressure. Br. J. Ophthalmol..

[62] Sugiyama T., Schwartz B., Takamoto T., Azuma I. (2000). Evaluation of the Circulation in the Retina, Peripapillary Choroid and Optic Disk in Normal-Tension Glaucoma. Ophthalmic Res..

[63] Park J.W., Kwon H.J., Chung W.S., Kim C.Y., Seong G.J. (2011). Short-Term Effects of Ginkgo biloba Extract on Peripapillary Retinal Blood Flow in Normal Tension Glaucoma. Korean J. Ophthalmol..

[64] Hamard P., Hamard H., Dufaux J., Quesnot S. (1994). Optic nerve head blood flow using a laser Doppler velocimeter and haemorheology in primary open angle glaucoma and normal pressure glaucoma. Br. J. Ophthalmol..

[65] Mann C., Anders F., Liu H., Brockhaus K., Liu A., Grus F.H., Pfeiffer N., Thanos S., Prokosch V. (2018). Erhöhter Augeninnendruck für 7 Wochen induziert lokale Gefäßveränderungen im experimentellen Glaukommodell In Vivo. Klin. Mon. Augenheilkd..

[66] Ruan Y., Jiang S., Musayeva A., Gericke A. (2020). Oxidative Stress and Vascular Dysfunction in the Retina: Therapeutic Strategies. Antioxidants.

[67] Koenitzer J.R., Isbell T.S., Patel H.D., Benavides G.A., Dickinson D.A., Patel R.P., Darley-Usmar V.M., Lancaster J.R., Doeller J.E., Kraus D.W. (2007). Hydrogen sulfide mediates vasoactivity in an O_2_-dependent manner. Am. J. Physiol. Circ. Physiol..

[68] Kulkarni-Chitnis M., Njie-Mbye Y.F., Mitchell L., Robinson J., Whiteman M., Wood M.E., Opere C.A., Ohia S.E. (2015). Inhibitory action of novel hydrogen sulfide donors on bovine isolated posterior ciliary arteries. Exp. Eye Res..

[69] Peng S.-Y., Wu X., Lu T., Cui G., Chen G. (2020). Research progress of hydrogen sulfide in Alzheimer’s disease from laboratory to hospital: A narrative review. Med Gas Res..

[70] Nagpure B.V., Bian J.-S. (2014). Hydrogen Sulfide Inhibits A2A Adenosine Receptor Agonist Induced β-Amyloid Production in SH-SY5Y Neuroblastoma Cells via a cAMP Dependent Pathway. PLoS ONE.

[71] Kumar M. (2018). Hydrogen Sulfide in Physiological and Pathological Mechanisms in Brain. CNS Neurol. Disord. Drug Targets.

[72] Liu X.Q., Liu X.Q., Jiang P., Huang H., Yan Y. (2008). Plasma levels of endogenous hydrogen sulfide and homo-cysteine in patients with alzheimer’s disease and vascular dementia and the significance thereof. Zhonghua Yi Xue Za Zhi.

[73] Eto K., Asada T., Arima K., Makifuchi T., Kimura H. (2002). Brain hydrogen sulfide is severely decreased in Alzheimer’s disease. Biochem. Biophys. Res. Commun..

[74] Gozzelino R., Arosio P. (2016). Iron Homeostasis in Health and Disease. Int. J. Mol. Sci..

[75] Gutteridge J.M.C., Rowley D.A., Halliwell B. (1981). Superoxide-dependent formation of hydroxyl radicals in the presence of iron salts. Detection of ’free’ iron in biological systems by using bleomycin-dependent degradation of DNA. Biochem. J..

[76] Liu H., Perumal N., Manicam C., Mercieca K., Prokosch V. (2020). Proteomics Reveals the Potential Protective Mechanism of Hydrogen Sulfide on Retinal Ganglion Cells in an Ischemia/Reperfusion Injury Animal Model. Pharmaceuticals.

[77] Schröder N., Figueiredo L.S., de Lima M.N.M. (2013). Role of Brain Iron Accumulation in Cognitive Dysfunction: Evidence from Animal Models and Human Studies. J. Alzheimer’s Dis..

[78] Bartzokis G., Lu P.H., Tingus K., Peters D.G., Amar C.P., Tishler T.A., Finn J.P., Villablanca P., Altshuler L.L., Mintz J. (2011). Gender and Iron Genes May Modify Associations Between Brain Iron and Memory in Healthy Aging. Neuropsychopharmacology.

[79] De Lima M.N.M., Polydoro M., Laranja D.C., Bonatto F., Bromberg E., Moreira J.C.F., Schröder N., Dal-Pizzol F. (2005). Recognition memory impairment and brain oxidative stress induced by postnatal iron administration. Eur. J. Neurosci..

[80] Fredriksson A., Schroder N., Eriksson P., Izquierdo I., Archer T. (1999). Neonatal Iron Exposure Induces Neurobehavioural Dysfunctions in Adult Mice. Toxicol. Appl. Pharmacol..

[81] Ward R.J., Zucca F.A., Duyn J.H., Crichton R.R., Zecca L. (2014). The role of iron in brain ageing and neurodegenerative disorders. Lancet Neurol..

[82] Nabuurs R.J.A., Hegeman I., Natté R., van Duinen S.G., van Buchem M.A., van der Weerd L., Webb A.G. (2010). High-field MRI of single histological slices using an inductively coupled, self-resonant microcoil: Application to ex vivo samples of patients with Alzheimer’s disease. NMR Biomed..

[83] Farkas R.H., Chowers I., Hackam A.S., Kageyama M., Nickells R.W., Otteson D.C., Duh E.J., Wang C., Valenta D.F., Gunatilaka T.L. (2004). Increased Expression of Iron-Regulating Genes in Monkey and Human Glaucoma. Investig. Opthalmol. Vis. Sci..

[84] Hohberger B., Chaudhri M.A., Michalke B., Lucio M., Nowomiejska K., Schlötzer-Schrehardt U., Grieb P., Rejdak R., Jünemann A.G. (2018). Levels of aqueous humor trace elements in patients with open-angle glaucoma. J. Trace Elem. Med. Biol..

[85] Goralska M., Ferrell J.B., Harned J., Lall M., Nagar S., Fleisher L.N., McGahan M.C. (2009). Iron metabolism in the eye: A review. Exp. Eye Res..

[86] Gozzelino R. (2016). The Pathophysiology of Heme in the Brain. Curr. Alzheimer Res..

[87] Barone E., Trombino S., Cassano R., Sgambato A., de Paola B., di Stasio E., Picci N., Preziosi P., Mancuso C. (2009). Characterization of the S-denitrosylating activity of bilirubin. J. Cell. Mol. Med..

[88] Crack J.C., Green J., Thomson A.J., le Brun N.E. (2014). Iron–Sulfur Clusters as Biological Sensors: The Chemistry of Reactions with Molecular Oxygen and Nitric Oxide. Acc. Chem. Res..

[89] Picard E., Daruich A., Youale J., Courtois Y., Behar-Cohen F. (2020). From Rust to Quantum Biology: The Role of Iron in Retina Physiopathology. Cells.

[90] Stemmler T.L., Lesuisse E., Pain D., Dancis A. (2010). Frataxin and Mitochondrial FeS Cluster Biogenesis. J. Biol. Chem..

[91] Lill R., Hoffmann B., Molik S., Pierik A.J., Rietzschel N., Stehling O., Uzarska M.A., Webert H., Wilbrecht C., Mühlenhoff U. (2012). The role of mitochondria in cellular iron–sulfur protein biogenesis and iron metabolism. Biochim. Biophys. Acta Bioenerg..

[92] Rouault T.A. (2014). Mammalian iron–sulphur proteins: Novel insights into biogenesis and function. Nat. Rev. Mol. Cell Biol..

[93] Lill R. (2009). Function and biogenesis of iron–sulphur proteins. Nat. Cell Biol..

[94] Cardenas-Rodriguez M., Chatzi A., Tokatlidis K. (2018). Iron–sulfur clusters: From metals through mitochondria biogenesis to disease. JBIC J. Biol. Inorg. Chem..

[95] Pandey A., Pain J., Ghosh A.K., Dancis A., Pain D. (2015). Fe-S Cluster Biogenesis in Isolated Mammalian Mitochondria. J. Biol. Chem..

[96] Qi W., Li J., Chain C.Y., Pasquevich G.A., Pasquevich A.F., Cowan J.A. (2012). Glutathione Complexed Fe–S Centers. J. Am. Chem. Soc..

[97] Kamel K., Farrell M., O’Brien C. (2017). Mitochondrial dysfunction in ocular disease: Focus on glaucoma. Mitochondrion.

[98] Tabassum R., Jeong N.Y. (2019). Potential for therapeutic use of hydrogen sulfide in oxidative stress-induced neurodegenerative diseases. Int. J. Med. Sci..

[99] Liu H., Mercieca K., Prokosch V. (2020). Mitochondrial Markers in Aging and Primary Open-Angle Glaucoma. J. Glaucoma.

[100] Reddy P.H. (2009). Role of Mitochondria in Neurodegenerative Diseases: Mitochondria as a Therapeutic Target in Alzheimer’s Disease. CNS Spectrums.

[101] Reddy P.H. (2009). Amyloid beta, mitochondrial structural and functional dynamics in Alzheimer’s disease. Exp. Neurol..

[102] Devi L., Prabhu B.M., Galati D.F., Avadhani N.G., Anandatheerthavarada H.K. (2006). Accumulation of Amyloid Precursor Protein in the Mitochondrial Import Channels of Human Alzheimer’s Disease Brain Is Associated with Mitochondrial Dysfunction. J. Neurosci..

[103] Coskun P.E., Beal M.F., Wallace D.C. (2004). Alzheimer’s brains harbor somatic mtDNA control-region mutations that suppress mitochondrial transcription and replication. Proc. Natl. Acad. Sci. USA.

[104] Osborne N.N. (2010). Mitochondria: Their role in ganglion cell death and survival in primary open angle glaucoma. Exp. Eye Res..

[105] Cadenas E., Davies K.J. (2000). Mitochondrial free radical generation, oxidative stress, and aging. Free Radic. Biol. Med..

[106] Hayakawa M., Sugiyama S., Hattori K., Takasawa M., Ozawa T. (1993). Age-associated damage in mitochondrial DNA in human hearts. Mol. Cell. Biochem..

[107] Völkel S., Grieshaber M.K. (1996). Mitochondrial Sulfide Oxidation in Arenicola Marina. Evidence for Alternative Electron Pathways. JBIC J. Biol. Inorg. Chem..

[108] Goubern M., Andriamihaja M., Nübel T., Blachier F., Bouillaud F. (2007). Sulfide, the first inorganic substrate for human cells. FASEB J..

[109] Hildebrandt T.M., Grieshaber M.K. (2008). Redox regulation of mitochondrial sulfide oxidation in the lugworm, Arenicola marina. J. Exp. Biol..

[110] Leschelle X., Goubern M., Andriamihaja M., Blottière H.M., Couplan E., Gonzalez-Barroso M.-D.-M., Petit C., Pagniez A., Chaumontet C., Mignotte B. (2005). Adaptative metabolic response of human colonic epithelial cells to the adverse effects of the luminal compound sulfide. Biochim. Biophys. Acta Gen. Subj..

[111] López-Lluch G., Irusta P.M., Navas P., de Cabo R. (2008). Mitochondrial biogenesis and healthy aging. Exp. Gerontol..

[112] Filler K., Lyon D., Bennett J., McCain N., Elswick R., Lukkahatai N., Saligan L.N. (2014). Association of mitochondrial dysfunction and fatigue: A review of the literature. BBA Clin..

[113] Szczesny B., Módis K., Yanagi K., Coletta C., le Trionnaire S., Perry A., Wood M.E., Whiteman M., Szabo C. (2014). AP39, a novel mitochondria-targeted hydrogen sulfide donor, stimulates cellular bioenergetics, exerts cytoprotective effects and protects against the loss of mitochondrial DNA integrity in oxidatively stressed endothelial cells in vitro. Nitric Oxide.

[114] Li S., Yang G. (2015). Hydrogen Sulfide Maintains Mitochondrial DNA Replication via Demethylation of TFAM. Antioxid. Redox Signal..

[115] Carter-Dawson L., Shen F., Harwerth R.S., Crawford M., Smith E.L., Whitetree A. (2004). Glutathione content is altered in Müller cells of monkey eyes with experimental glaucoma. Neurosci. Lett..

[116] Deleve L.D., Kaplowitz N. (1991). Glutathione metabolism and its role in hepatotoxicity. Pharmacol. Ther..

[117] Schulz J.B., Lindenau J., Seyfried J., Dichgans J. (2000). Glutathione, oxidative stress and neurodegeneration. JBIC J. Biol. Inorg. Chem..

[118] Harada T., Harada C., Nakamura K., Quah H.-M.A., Okumura A., Namekata K., Saeki T., Aihara M., Yoshida H., Mitani A. (2007). The potential role of glutamate transporters in the pathogenesis of normal tension glaucoma. J. Clin. Investig..

[119] Schubert D., Piasecki D. (2001). Oxidative Glutamate Toxicity Can Be a Component of the Excitotoxicity Cascade. J. Neurosci..

[120] Kimura Y., Goto Y.-I., Kimura H. (2010). Hydrogen Sulfide Increases Glutathione Production and Suppresses Oxidative Stress in Mitochondria. Antioxid. Redox Signal..

[121] Chan J.Y., Kwong M. (2000). Impaired expression of glutathione synthetic enzyme genes in mice with targeted deletion of the Nrf2 basic-leucine zipper protein. Biochim. Biophys. Acta Gene Struct. Expr..

[122] Majid A.S.A., Majid A.M.S.A., Yin Z.Q., Ji D. (2013). Slow Regulated Release of H2S Inhibits Oxidative Stress Induced Cell Death by Influencing Certain Key Signaling Molecules. Neurochem. Res..

